# Impact of the Lipopolysaccharide Chemotype of *Salmonella Enterica* Serovar Typhimurium on Virulence in Gnotobiotic Piglets

**DOI:** 10.3390/toxins11090534

**Published:** 2019-09-13

**Authors:** Alla Splichalova, Zdislava Splichalova, Daniela Karasova, Ivan Rychlik, Paolo Trevisi, Marek Sinkora, Igor Splichal

**Affiliations:** 1 Laboratory of Gnotobiology, Institute of Microbiology, Czech Academy of Sciences, 549 22 Novy Hradek, Czech Republic; splichalova@gnotobio.cz (A.S.); zdispl@gnotobio.cz (Z.S.); marek@biomed.cas.cz (M.S.); 2 Department of Immunology, Veterinary Research Institute, 621 00 Brno, Czech Republic; karasova@vri.cz (D.K.); rychlik@vri.cz (I.R.); 3 Department of Agricultural and Food Sciences, University of Bologna, 40127 Bologna, Italy; paolo.trevisi@unibo.it

**Keywords:** lipopolysaccharide, *Salmonella* Typhimurium, Δ*rfa* mutant, cytokines, tight junction proteins, gnotobiotic, germ-free piglet

## Abstract

*Salmonella* Typhimurium is an enteric pathogen that causes acute and chronic infections in humans and animals. One-week-old germ-free piglets were orally colonized/infected with the *Salmonella* Typhimurium LT2 strain or its isogenic rough Δ*rfaL*, Δ*rfaG* or Δ*rfaC* mutants with exactly defined lipopolysaccharide (LPS) defects. After 24 h, the piglets were euthanized and the colonization of the small intestine, translocations into the mesenteric lymph nodes, liver, spleen, lungs, and bacteremia, along with changes in the ileum histology, and transcription levels of the tight junction proteins claudin-1, claudin-2, and occludin were all assessed. Additionally, transcription levels of IL-8, TNF-α, and IL-10 in the terminal ileum, and their local and systemic protein levels were evaluated. Wild-type *Salmonella* Typhimurium showed the highest translocation, histopathological changes, upregulation of claudins and downregulation of occludin, transcription of the cytokines, intestinal IL-8 and TNF-α levels, and systemic TNF-α and IL-10 levels. Depending on the extent of the incompleteness of the LPS, the levels of the respective elements decreased, or no changes were observed at all in the piglets colonized/infected with Δ*rfa* mutants. Intestinal IL-10 and systemic IL-8 levels were not detected in any piglet groups. This study provided foundational data on the gnotobiotic piglet response to colonization/infection with the exactly defined rough *Salmonella Typhimurium* LT2 isogenic mutants.

## 1. Introduction

Members of the genus *Salmonella* are enteric pathogens that cause acute and chronic infections in a broad range of hosts [1]. *Salmonella enterica* serovar Typhimurium (*Salmonella* Typhimurium) belongs among the most frequent non-typhoid *Salmonella* serovars that cause gastroenteritis in both humans and pigs [2,3]. While it can cause self-limited gastroenteritis in healthy individuals, infection with *Salmonella* Typhimurium can progress to a life-threatening systemic illness in immunocompromised patients [4,5].

Lipopolysaccharide (LPS) is a prominent virulence factor of Gram-negative bacteria and is the most abundant component of their cell walls. It forms a selectivity permeable barrier that restricts the entry of molecules into the bacterial cell [6] and it is composed of the lipid A, the core oligosaccharide, and the O-antigen consisting of repeating sugar units [7]. LPS can be released either from damaged Gram-negative bacteria cell wall or via outer membrane vesicles [8]. It triggers the host innate immune response during infection through recognition of the lipid A (endotoxin) by Toll-like receptor 4 complex. This initiates a signal cascade leading to production of cytokines that are crucial for clearance of infection [7]. Endotoxemia can be manifested from imperceptible dysregulation of bioactive substances [9] to life-endangering multiple organ failure induced by exaggerated levels of inflammatory mediators [10]. A completely synthesized LPS is known as the S-form (smooth). LPS may also be present in incomplete forms as semi-rough (SR) and rough (R) in the order of decreasing completion of the core oligosaccharide [11]. Constructions and experimental use of defined *Salmonella enterica* LPS mutants as safe oral vaccine candidates in conventional mice [12] and pigs [13] were reported. In these experiments, attention was paid to the induction of specific immunity. The innate immune response, however (a first sentinel of immune defense), was studied with partially characterized rough *Salmonella enterica* mutants only [14,15].

This study aimed to determine the role of specific parts of LPS, such as O-antigen, and the outer and inner core in the innate immune response and pathological changes in the gnotobiotic piglet ileum without bacterial interferences of non-defined conventional microbiota. For this purpose, we used *Salmonella* Typhimurium mutants with decreasing completeness of the LPS, in the direction of wild-type > Δ*rfaL* > Δ*rfaG* > Δ*rfaC*. We have shown previously that these mutations attenuated strains of serovars Typhimurium and Enteritidis for chickens. Moreover, these mutants differentially interacted with porcine leukocytes and exhibited differential protein secretion in vitro [16,17,18,19]. We were therefore interested in the interaction of the Δ*rfa* mutants with an extremely sensitive model represented by germ-free piglets.

Pigs are used as an animal model in biomedical research studies due to their closely related anatomy, genetics, and physiology to humans [20], and represent a suitable animal model of human infectious diseases [21]. Moreover, the sensitivity of the pig to LPS is similar to that of humans [22] in contrast to rodents that are much more resistant [23]. Gnotobiotic animals with lowered colonization resistance [24] make it possible to study host interactions with less virulent microbes that could be suppressed in the presence of a balanced microbiota [25,26]. Colostrum-free piglets, deprived of maternal immunoglobulins and cells [27] and reared in a microbiologically controlled (gnotobiotic) isolator, can be used as a model of immunocompromised infants [28]. *Salmonella* Typhimurium strain LT2 [29] is known as “laboratory strain”. It induced a weak inflammatory response in the intestine of one-week-old conventional piglets [30], but it caused a strong response in germ-free piglets [31].

We hypothesized that the virulence of different rough mutants would decrease with decreasing completeness of the LPS, as found in the Δ*rfa* mutants of *Salmonella* Typhimurium strain *X* 9241 in conventional BALB/c mice [12]. However, *Salmonella* Typhimurium causes in conventional mice illness similar to typhoid fever in contrast to gastroenteritis in humans and pigs [32]. To test our hypothesis, we orally colonized/infected one-week-old hysterectomy-derived colostrum-deprived germ-free piglets with the *Salmonella* Typhimurium LT2 strain or its isogenic Δ*rfaL*, Δ*rfaG*, and Δ*rfaC* mutants for 24 h.

## 2. Results

### 2.1. Clinical Signs

All piglets survived for the experimental period of 24 h. The germ-free piglets inoculated with wild-type *Salmonella* Typhimurium developed diarrhea 10 h post-infection, and their feces changed from pale brown and pasty, to yellow and watery. They also suffered from fever, anorexia, somnolence, tachycardia, tachypnea, and tremor. The piglets infected with the Δ*rfaL* mutant showed similar signs. Weaker and delayed signs were expressed in the Δ*rfaG*-infected group. The piglets inoculated with the Δ*rfaC Salmonella* Typhimurium mutant less expressed clinical signs of salmonellosis than the Δ*rfaG* piglets.

### 2.2. Salmonella Colonization of the Small Intestine and Its Translocation

**Figure 1:**
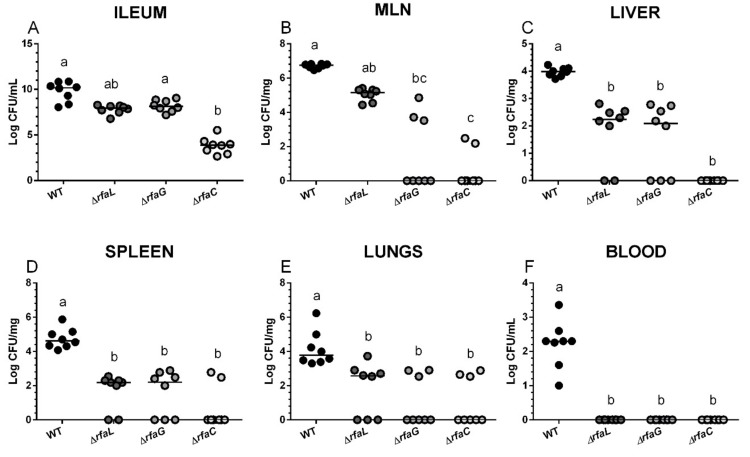
Bacterial counts in the small intestine, mesenteric lymph nodes, liver, spleen, lungs, and blood of the gnotobiotic piglets. *Salmonella* Typhimurium (WT) and its isogenic Δ*rfaL*, Δ*rfaG*, and Δ*rfaC* mutant colony forming units (CFU) were counted in the ileum (**A**), mesenteric lymph nodes (MLN) (**B**), the liver (**C**), the spleen (**D**), the lungs (**E**), and the blood (**F**) 24 h post infection. Individual log CFU counts are depicted by circles (*n* = 8 in each group) and the median by a horizontal line. The Kruskal-Wallis test with Dunn’s post-hoc multiple comparisons test was used, and statistical differences *p* < 0.05 are denoted with different letters above the groups. The same letter above the groups indicates no statistically significant differences.

Wild-type *Salmonella* Typhimurium colonized the small intestine at the highest degree, with a median count greater than 10^10^ CFU/g in the ileum (Figure 1A). Δ*rfaL* and Δ*rfaG* mutants colonized the intestine at a lower density with medians around 10^8^ CFU/g, and this decrease was statistically significant in Δ*rfaL* but not in the Δ*rfaG* mutant. Colonization ability of the Δ*rfaC* mutant was 6-log lower in comparison to the wild-type *Salmonella* Typhimurium, and 4-log lower as compared to the Δ*rfaG* mutant. Wild-type *Salmonella* Typhimurium was found in all tested organs of all piglets—mesenteric lymph nodes (Figure 1B), liver (Figure 1C), spleen (Figure 1D), and lungs (Figure 1E), and caused bacteremia (Figure 1F). *Salmonella* Typhimurium counts in mesenteric lymph nodes (MLN) (Figure 1B) are directly proportional to the completeness of the LPS. Translocations of Δ*rfaG*, and Δ*rfaC* mutants, but not Δ*rfaL*, were significantly lowered. Three out of 8 Δ*rfaG* and 2 out of 6 Δ*rfaC*, respectively, were found in the MLN of these groups. The liver translocation (Figure 1C) was detected in all WT-infected piglets, but only in 6, 5, and 0 out of 8 piglets infected with the Δ*rfaL*, Δ*rfaG*, and Δ*rfaC* mutants, respectively. Similarly, the wild-type *Salmonella* Typhimurium infected the spleen of all piglets (Figure 1D), while Δ*rfaL*, Δ*rfaG*, and Δ*rfaC* mutants were detected in only 6, 5 and 2 out of 8 infected piglets, respectively. All the mutants displayed low levels of lung translocation (Figure 1E).

### 2.3. Histopathological Changes in the Ileum

**Figure 2:**
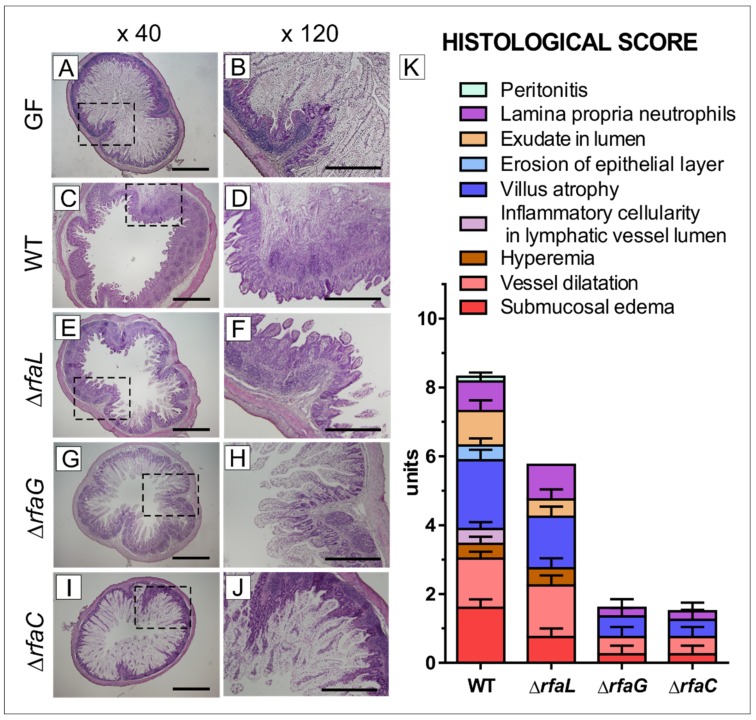
Representative hematoxylin and eosin-stained cross sections of the ileum in the gnotobiotic piglets and a histological score. The ileum of one-week-old gnotobiotic piglets: germ-free piglets (GF; **A**,**B**), piglets colonized/infected with *Salmonella* Typhimurium strain LT2 for 24 hrs (WT; **C**,**D**) or its isogenic mutants Δ*rfa*L (**E**,**F**), Δ*rfa*G (**G**,**H**) or Δ*rfa*C (**I**,**J**). Bars represent 1 mm (**A**,**C**,**E**,**G**,**I**) and 500 μm (**B**,**D**,**F**,**H**,**J**) cross sections, respectively. Histological scores from the ileum of six piglets per group are depicted (**K**).

The ileum of the germ-free piglets showed long villi containing abundant vacuolated enterocytes and no signs of inflammation (Figure 2 A,B). In contrast, the ileum of the piglets infected with wild-type *Salmonella* Typhimurium (Figure 2C,D) was affected by acute inflammation characterized by villus atrophy, submucosal edema, vessel dilation, exudate in the lumen, the presence of neutrophils in the lamina propria, hyperemia, erosion of the epithelial layer, and peritonitis, as it is summarized in the histological score (Figure 2K). The majority of these changes, expressed at lower levels, were also found in the Δ*rfaL* group (Figure 2E,F,K). Hemorrhage was absent in the WT and other groups. Inflammatory changes in the Δ*rfaG* (Figure 2G,H) and Δ*rfaC* (Figure 2I,J) groups were indiscernible—with only negligible villus atrophy, vessel dilation, neutrophils in the lamina propria, and submucosal edema. Using our scoring system, the total histological score for WT, Δ*rfaL*, Δ*rfaG*, and Δ*rfaC* groups were evaluated as 8.2, 5.8, 1.9, and 1.9, respectively (Figure 2K).

### 2.4. Expression of Tight Junction Proteins Claudin-1, Claudin-2, and Occludin in the Ileum

**Figure 3:**
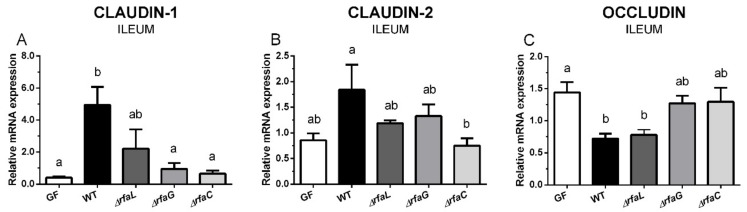
Transcription levels of claudin-1, claudin-2, and occludin in the ileum of the gnotobiotic piglets. Claudin-1 (**A**), claudin-2 (**B**), and occludin (**C**) mRNA were normalized to β-actin and cyclophilin A. The relative expressions (fold change) were evaluated in the germ-free piglets (GF) and the piglets colonized/infected with wild-type *Salmonella* Typhimurium (WT), or its isogenic Δ*rfaL*, Δ*rfaG*, and Δ*rfaC* mutants. One-way analysis of variance (ANOVA) with Tukey’s multiple comparisons post-hoc test was used to compare differences among the groups. The values are presented as mean + SEM. Statistical differences *p* < 0.05 are denoted with different letters above the columns, and the same letter shown above the column indicates no statistically significant differences. Six samples in each group were analyzed.

The transcription of claudin-1 increased after the infection of the germ-free piglets with the wild-type *Salmonella* Typhimurium and the Δ*rfaL* mutant (Figure 3A). Statistical differences were observed between the WT, and the germ-free, Δ*rfaG*, and Δ*rfaC* groups. Claudin-2 transcription was increased in the WT group, and statistical differences were found between this group and the Δ*rfaC* group, but not other piglets (Figure 3B). Occludin (Figure 3C) showed the opposite trend than the claudins (Figure 3A,B), i.e., its transcription in the ileum of wild-type and Δ*rfaL Salmonella* Typhimurium-infected piglets was statistically significantly lower than in germ-free piglets. Comparable relations to other groups were found in the Δ*rfaL* mutant–infected piglets. However, no statistical differences were found between WT or Δ*rfaL* groups, or between the Δ*rfaG* or Δ*rfaC* groups.

### 2.5. Transcriptions of IL-8, TNF-α, and IL-10 in the Ileum, and Their Local and Systemic Levels

**Figure 4:**
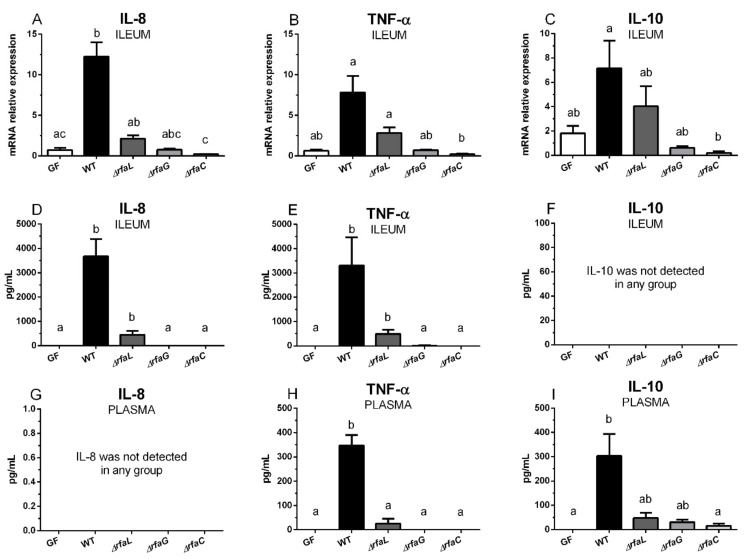
Transcription levels of IL-8, TNF-α, and IL-10 in the ileum tissue and their protein levels in the ileum lavage and plasma. Transcriptions of IL-8 (**A**), TNF-α (**B**), and IL-10 (**C**) in the terminal ileum tissue of the germ-free piglets (GF) and the piglets colonized/infected with wild-type *Salmonella* Typhimurium (WT), or its isogenic Δ*rfaL*, Δ*rfaG*, and Δ*rfaC* mutants, were normalized to β-actin and cyclophilin A. The protein levels of the same cytokines—IL-8 (**D**), TNF-α (**E**), and IL-10 (**F**)—in the ileum lavage and the plasma (**G**,**H**,**I**, respectively) were estimated. Kruskal-Wallis test with Dunn’s multiple comparison post-hoc test was used to compare the groups. The transcriptions (**A**–**C**) were measured in six piglets per group, and the protein levels (**D**–**I**) in eight piglets per group. The values are presented as mean + SEM. Statistical differences *p* < 0.05 are denoted with different letters above the columns and the same letter shown above the column indicates no statistically significant differences.

Wild-type *Salmonella* Typhimurium induced transcriptions of IL-8 (Figure 4A), TNF-α (Figure 4B), and IL-10 (Figure 4C) in the ileum, as compared to the control germ-free piglets. Only in the case of IL-8 was this induction statistically significant. IL-8 (Figure 4D) and TNF-α (Figure 4E) levels were significantly induced by wild-type *Salmonella* Typhimurium, and its Δ*rfaL* mutant, in the ileum. No IL-10 levels in the ileum (Figure 4F) or IL-8 levels in plasma (Figure 4G) were found. TNF-α in plasma (Figure 4H) was induced by wild-type *Salmonella* Typhimurium and was statistically different from other groups. Similarly, IL-10 was increased in plasma (Figure 4I) and was statistically different in the Δ*rfaC* and germ-free groups, but not in the Δ*rfaL* and Δ*rfaG* groups.

## 3. Discussion

*Salmonella* translocates beyond the intestinal barrier to the mesenteric lymph nodes (MLN) via lymphatic vessels, while less virulent strains can be trapped and destroyed [33]. Highly virulent *Salmonella* can spread via lymph vessels to the liver, spleen and other organs, or it can reach these sites via the blood [34]. *Salmonella* Typhimurium strain LT2 is avirulent for conventional piglets [30], but the germ-free piglets infected with this strain die 36–48 hrs after the infection (unpublished results). While wild-type *Salmonella* Typhimurium LT2 translocated to all observed organs and caused bacteremia in our experiments, its Δ*rf*a mutants with incompletely synthetized LPS showed lowered or neglected translocation and did not cause bacteremia. These findings corresponded to the findings in conventional mice [12].

Wild-type *Salmonella* Typhimurium LT2 and the Δ*rfaL* mutant induced devastating histopathological changes in the ileum of the full-term gnotobiotic piglets, but with no hemorrhages, unlike the previous study performed in preterm hysterectomy-derived gnotobiotic piglets [28]. The mutants with more incomplete LPS chains (Δ*rfaG* and Δ*rfaC*) showed a reduced detrimental effect, as did other rough *Salmonella* strains of different serovars [14,33].

The intestinal epithelium, the single cell layer of epithelial cells in the gastrointestinal tract, is important for both nutrient uptake and protection of the host against bacterial translocation. The barrier function is dependent on tight junction proteins such as claudins and occludin, which join adjacent enterocytes in the apical region. This barrier can be disturbed with enteric infections [35]. Claudin-1 belongs to the group of barrier-forming claudins, and claudin-2 to the pore-forming claudins [36]. The increase in claudin-1 transcription in the ileum of the wild-type *Salmonella* Typhimurium-infected gnotobiotic piglets probably indicates the body’s attempt to seal the intestinal barrier to protect the piglets against excessive loss of electrolytes via diarrhea. The scope of claudin-1 induction gradually decreased in piglets infected with Δ*rfa* mutants, with the decreasing completeness of their lipopolysaccharide chains in the direction of Δ*rfaL* > Δ*rfaG* > Δ*rfaC,* corroborating with decreasing severity of the infection as evaluated by clinical signs. In contrast to our finding, the conventional piglet ileum infected with *Salmonella* Infantis showed decreased claudin-1 protein [37]. This discrepancy should be explained by the attempt to seal the intestinal barrier from the beginning of infection, but by manifestation of its devastating effect five days post infection. Claudin-2 was increased in the ST group too. We suppose that these changes of claudin-2 with seemingly opposite function than claudin-1 [36] complete the attempt to maintain the functionality of damaged intestinal barrier. In contrast, transcription of occludin decreased in wild-type *Salmonella* Typhimurium and Δ*rfaL* mutant-infected piglets. Although occludin is also a tight junction protein and is related to the transfer of large biomolecules and cells [38], its function is much less understood than that of the claudins [36]. We were unaware of a precise mechanism of action by *Salmonella* Typhimurium that resulted in occludin’s expression opposite to that reported for claudins, but *Salmonella* Typhimurium alters the tight junctions in the epithelial barrier that result in increased bacterial translocation and induction of neutrophil transepithelial migration [39]. Similarly, neutrophil recruitment into inflamed intestine was found in *Salmonella* Typhimurium-induced colitis in streptomycin-treated C57Bl/6 mice [40]. Our newly obtained data are in agreement with previous findings in preterm gnotobiotic piglets colonized with probiotic *Lactobacillus rhamnosus* GG, and challenged with virulent *Salmonella* Typhimurium [41]. Other authors found that the expression of occludin protein in the conventional *Salmonella* Infantis-infected piglets was not influenced five days after challenge [37].

Inflammatory changes are governed by inflammatory mediators, mainly by cytokines. *Salmonella* Typhimurium can disrupt tight junctions, either by direct contact with epithelium or via cytokine induction [42]. To monitor piglet response to *Salmonella* Typhimurium challenge, we used three cytokines with different activities—chemotactic cytokine (chemokine) interleukin IL-8, a pro-inflammatory cytokine TNF-α, and a regulatory cytokine IL-10 [43]. IL-8 attracts neutrophils into inflammatory site [44], TNF-α is a pluripotent cytokine that is a potent mediator of inflammation inducible by LPS [45], and IL-10 regulates immune response to prevent an excessive inflammatory reaction [46]. Gene transcripts of all three cytokines increased in the terminal ileum of the piglets infected with wild-type *Salmonella* Typhimurium. Expression of these cytokines was also partially induced by the Δ*rfaL* mutant, while infection of piglets with Δ*rfaG* and Δ*rfaC* did not induce expression of these cytokines above the levels observed in the non-infected controls. These results, together with the above-mentioned translocations and histological changes, indicate high virulence and the stimulatory effect of wild-type *Salmonella* Typhimurium and decreasing virulence with the decreasing completeness of the LPS chains. This is in agreement with in vitro observations in which the Δ*rfaC* mutant manifested lower protein secretion and invasion compared to wild-type *Salmonella* Typhimurium. So the total virulence of the *Salmonella enterica* mutants with affected LPS might be a sum of different structure, different length and other affected processes like protein secretion and motility [16].

Only a few data are available that characterize the presence of inflammatory cytokines in intestinal content during enteric infections. Jeong et al. found significantly elevated IL-8, TNF-α, and IL-10 levels in the jejunum and ileum of gnotobiotic piglets infected with *Shigella dysenteriae* at 24 h post-infection [47]. Our finding of increased levels of IL-8 in the distal small intestine corresponds to the presence of neutrophils in the ileum of both wild-type *Salmonella* Typhimurium and Δ*rfaL* mutant-infected piglets. We propose that high local levels of IL-8, together with high levels of TNF-α in the intestine, in the case of wild-type *Salmonella* Typhimurium, exceeded normal physiological levels and contributed to the damage of the intestine itself. Lower levels of IL-8 and TNF-α in the Δ*rfaL* group may correspond to the protective chemoattraction of neutrophils to the inflammatory site [14] as a mechanism of protection by rough *Salmonella enterica* strains against subsequent infection with virulent *Salmonella* Typhimurium [14,15,33]. Less understandable is the absence of secreted IL-10 in the ileum of all groups, since its transcription was highly induced in wild-type *Salmonella* Typhimurium and Δ*rfaL* mutant-infected piglets. A hypothesis on the possible immaturity of the intestine and absence of cells able to secrete IL-10, or the strong degrading effect of protease-rich surroundings can be excluded because IL-10 was found in the intestine of gnotobiotic piglets of the same age infected with *E. coli* O55 [48]. We propose that the secretion of IL-10 in the intestine might be delayed, and measurable later than 24 hrs post-infection. This delay is probably the reason why local IL-10 did not play a role in the regulation of the inflammatory response and did not ameliorate a cytokine storm and its deleterious consequences [10].

Increased mRNA levels for IL-8 and TNF-α in different parts of the intestine, in the conventional pig infected with *Salmonella* Typhimurium DT104, corresponded to the concurring serum values and showed significant increases with the highest levels at 1 dpi for IL-8 and 2 dpi for TNF-α [49]. Other conventional pigs infected with *Salmonella* Typhimurium X4232 showed increased serum levels of IL-8, TNF-α and IL-10 two days post-infection [50]. Systemic IL-8 was not found in blood plasma of the gnotobiotic piglets infected with *Salmonella* Typhimurium strain LT2 or colonized with probiotic *E. coli* Nissle O55 or avirulent *E. coli* O86. However, high systemic IL-8 levels were found in the piglets with *E. coli* O55 infection [48,51]. In our present study, we did not detect IL-8 in the plasma of the piglets infected with wild-type *Salmonella* Typhimurium or any of the Δ*rfa* mutants. This likely supports our supposition that longer time would be needed for the development of detrimental consequences of the infection with the virulent *Salmonella* Typhimurium, as we concluded in the case of IL-10 in the intestinal content.

Both TNF-α and IL-10 were highly increased in the plasma of wild-type *Salmonella* Typhimurium-infected piglets. High levels of systemic IL-10 and TNF-α indicated a poor prognosis of survival in preterm infants [52] and adult patients [53]. Similarly, in the gnotobiotic piglets, both IL-10 and TNF-α were found in *E. coli* O55 infected piglets ante finem [48]. High levels of IL-10 were detected in the piglets infected with wild-type *Salmonella,* and in lower levels in the plasma of the piglets infected with the Δ*rfa* mutants. They were absent, however, in the plasma of the control germ-free piglets. This further contrasted with the absence of IL-10 in the intestinal lavages, and the source of the plasma IL-10 is currently unknown.

## 4. Conclusions

In this study, we characterized the course of infection of germ-free piglets with wild-type and Δ*rfa* mutants of *Salmonella* Typhimurium. Any defect in the LPS structure correlated with reduced virulence. However, the absence of O-antigen in the Δ*rfaL* mutant resulted in reduced, but still detectable, residual virulence. The absence of an outer core of LPS in both Δ*rfaG* and Δ*rfaC* mutants resulted in complete avirulence. This also means that additional defects in the structure of the inner core of LPS present in Δ*rfaC* resulted in no further reduction in virulence, even in an extremely sensitive model of colostrum-deprived germ-free piglets. These data will be used to plan future studies in gnotobiotic piglets colonized/infected for a longer period with wild-type *Salmonella* Typhimurium LT2 and its Δ*rfa* mutants, to determine suitable candidates for the induction of innate and specific immune response and protection of the piglets against subsequent infection with wild-type *Salmonella* Typhimurium.

## 5. Materials and Methods

### 5.1. Ethical Statement

All experiments with animals were approved by the Animal Care and Use Committee of the Czech Academy of Sciences of the Czech Republic (protocol #63/2015; date of approval: 6 September, 2015).

### 5.2. Salmonella Typhimurium LT2 Strain and Its Isogenic Δrfa Mutants

**Figure 5:**
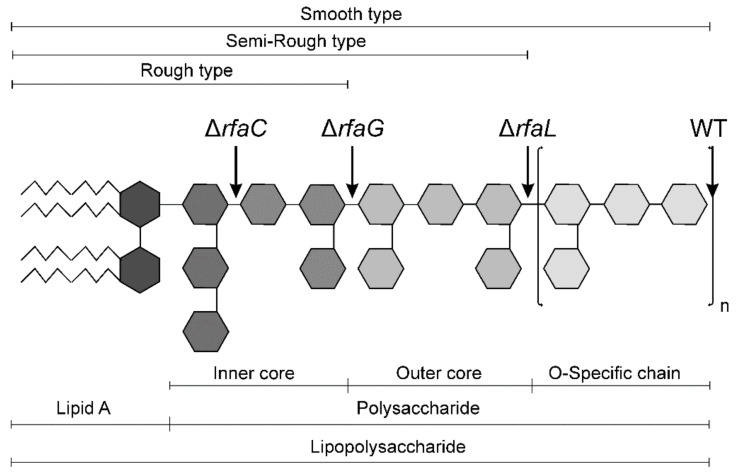
General structure of lipopolysaccharide [7,11]. The complete lipopolysaccharide (LPS) chain corresponds to the wild-type *Salmonella* Typhimurium (WT). The incompleteness of the LPS chain and corresponding chemotypes of the Δ*rfaC,* Δ*rfaG, and* Δ*rfaL* mutants are depicted.

The wild-type LT2 strain of *Salmonella enterica* serovar Typhimurium (WT) and its isogenic Δ*rfaL*, Δ*rfaG*, and Δ*rfaC* mutants with various completeness of LPS chain (Figure 5) were obtained from a collection of microorganisms of the Institute of Microbiology (Novy Hradek, Czech Republic). The mutants with different LPS chemotype were prepared by the same methods as described elsewhere [17] (Appendix A).

The bacterial inocula were prepared by the cultivation of bacteria on meat peptone agar slopes (blood agar base; Oxoid, Basingstoke, UK) at 37 °C overnight. The bacteria were taken from the agar slopes and resuspended in PBS to an approximate density of 5 × 10^8^ colony forming units CFU/mL. The number of CFU estimated by spectrophotometry at 600 nm was verified by a cultivation method on Luria-Bertani agar (Difco Laboratories, Detroit, MI, USA) at 37 °C for 24 h. The growth of the wild-type *Salmonella* Typhimurium and its Δ*rfa* mutants on the agar was comparable.

### 5.3. Gnotobiotic Piglets

Miniature Minnesota-derived germ-free piglets were obtained by hysterectomy as described elsewhere [28]. Briefly, hysterectomies were performed on the 112^th^ day of gestation under isoflurane (Isoflurane; Piramal Healthcare UK, Morpeth, UK) anesthesia. The total number of 40 gnotobiotic piglets were divided into five groups with eight piglets per group. Each piglet group was created from three independent hysterectomies. The piglet groups were reared separately in positive-pressure sterile fiberglass isolators with heated floors. They were fed to satiety 6–7 times a day with an autoclave-sterilized, condensed cow’s milk-based formula (Mlekarna Hlinsko, Hlinsko, Czech Republic), using a bottle with a nipple. The following were sampled and cultivated for the presence of aerobic and anaerobic bacteria, and mold: amniotic membranes, umbilical cords, meconium, mouth and isolator surface smears after hysterectomy and later mouth, isolator surface smears, and stool twice a week. Additionally, Gram-stained rectal swabs were inspected under a light microscope.

### 5.4. Colonization/Infection of the Germ-Free Piglets

One-week-old germ-free piglets were orally administered 1 × 10^8^ CFU of *Salmonella* Typhimurium (WT) or its isogenic LPS deletion mutants (Δ*rfaL*, Δ*rfaG*, and Δ*rfaC*) in 5 mL of milk diet. The control germ-free piglets received 5 mL of milk without *Salmonella*. Each group of piglets was created from three hysterectomies. Twenty-four h after the challenge, the piglets were euthanized, bled out via cardiac puncture under isoflurane anesthesia, and samples were collected.

### 5.5. Clinical Signs

The piglets were observed for the appearance of fever, anorexia, somnolence, and diarrhea.

### 5.6. Bacterial Colonization and Translocation

Samples of peripheral blood were cultivated both undiluted and as serial log-dilutions with PBS. Ileum lavage was obtained by cutting off a 40-cm segment of a terminal part of the small intestine containing the whole ileum and part of the jejunum, filling it with 2 mL of Dulbecco’s PBS (Life Technologies, Carlsbad, CA, USA), followed by gentle kneading, and rinsing. The vortexed intestinal lavages were diluted logarithmically in PBS. One g of each type of tissue (mesenteric lymph nodes, the liver, spleen, and lungs, respectively) was homogenized in 4 mL of deionized water in a glass piston homogenizer and serially diluted in PBS, to allow for separate analyses. The serial dilutions were aerobically cultivated on plates with LB agar (Difco Laboratories) at 37 °C for 24 h. The CFU were counted from dishes containing less than 200 colonies.

### 5.7. Blood Plasma and Intestinal Lavages

Citrated blood was spun at 1200× *g* for 10 min at 8 °C, and protease inhibitor cocktail (Roche Diagnostics, Manheim, Germany) was added to the collected plasma. The ileum lavages with added protease inhibitor cocktail (Roche Diagnostic) were spun at 2500× *g* for 30 min at 8 °C and supernatants were filtered through 0.2 μm nitrocellulose filters (Sartorius, Goettingen, Germany). Both the plasma and the lavage supernatants were immediately frozen and stored at −45 °C until the cytokines were measured.

### 5.8. Histologic Assessment

Terminal ileum samples were fixed in Carnoy’s fluid for 30 min, dehydrated and embedded in paraffin. Five μm tissue sections were cut on a Leica microtome RM2245 (Leica Microsystems, Wetzlar, Germany), stained with hematoxylin-eosin and examined under an Olympus BX 40 microscope with an Olympus Camedia C-2000 digital camera (Olympus, Tokyo, Japan). Sections were evaluated in a blinded fashion. The histological scoring was adapted from experiments with preterm piglets [41]: (i) submucosal edema (0–2 score points), (ii) polymorphonuclear neutrophils infiltration into the lamina propria (0–2 score points), (iii) villus atrophy (0–3 score points), (iv) exudate in lumen (0–2 score points), (v) vessel dilation (0–2 score points), vi) inflammatory cellularity in lymphatic vessel lumen (0–2 score points), (vii) hyperemia (0–2 score points), (viii) hemorrhage (0–2 score points), (ix) peritonitis (0–1 score points), and (x) erosion of the epithelial layer (0–3 score points). Total scores of 0–21 points were obtained.

### 5.9. Isolation of Total RNA and Reverse Transcription

1–2 mm-thin cross sections of the terminal ileum were placed in RNAlater (Qiagen, Hilden, Germany), and stored at −20 °C until RNA purification was performed. A Teflon piston homogenizer (Institute of Microbiology of the CAS, Novy Hradek, Czech Republic) and a Spin Tissue RNA Mini Kit (Stratec Molecular, Berlin, Germany) were used for total RNA purification according to the manufacturer’s instructions. Five hundred ng of the total RNA, with ratio absorbance at 260 and 280 nm ≥2.0 as measured in 10 mM Tris-HCl buffer (pH 7.5), were used for reverse transcription with the QuantiTect Reverse Transcription kit (Qiagen). The RNA was incubated at 42 °C for 2 min with a genomic DNA wipeout buffer. The synthesis of cDNA with a mixture of random hexamers and oligo d(T) primers was performed at 42 °C for 20 min, and then heated at 95 °C for 3 min. 180 µl of PCR quality water (Life Technologies, Carlsbad, CA, USA) was added to 20 μl of the synthetized cDNA mixture to prepare the PCR template, which was stored at −25 °C until Real-Time PCR was performed.

### 5.10. Real-Time PCR

To quantify specific sequences in the cDNA templates, 2 µL of cDNA template was added to 18 µL of the FastStart Universal Probe Master, which contained 100 nM LNA probe (both Roche Diagnostic, Manheim, Germany) and 500 nM each of the forward and reverse primers (Generi-Biotech, Hradec Kralove, Czech Republic) (Table 1). A heating protocol (95 °C for 10 min (1×), 95 °C for 15 s and 60 °C for 60 s (45×)) was used in the iQ5 Real-Time PCR cycler with iQ5 Optical System Software 1.0 (Bio-Rad, Hercules, CA, USA). The Cq of claudin-1, claudin-2, occludin, IL-8, TNF-α, and IL-10 was measured in duplicate, and the obtained values were normalized to the β-actin and cyclophilin A. Relative expressions were calculated by 2^−ΔCT^ method [54] using GenEx Pro 6 software (Multid Analyses AB, Gothenburg, Sweden).

### 5.11. ELISA

IL‑8 was detected as described elsewhere [55], while TNF-α and IL-10 were measured by commercial kits (Life Technologies, Carlsbad, CA, USA). IL-8, TNF-α, and IL-10 were measured with the sensitivities 20 pg/mL, 15 pg/mL, and 15 pg/mL, respectively. The assays were measured in two dilutions in duplicate at 450 nm and 620 nm with the Infinite M200 microplate reader (Tecan, Grodig, Austria), and results were evaluated with Magellan 6.3 software (Tecan, Grodig, Austria).

### 5.12. Statistical Analysis

The counts of wild-type *Salmonella* Typhimurium and its isogenic Δ*rfa* mutants in the small intestine, their translocations into different organs, and the cytokine transcription and protein levels were compared among the groups with a Kruskal-Wallis multiple comparisons test with a post-hoc Dunn’s test. One-way analysis of variance (ANOVA) with Tukey’s post-hoc test was used in the evaluation of differences in the tight junction protein transcription levels. The statistical comparisons at *p* < 0.05 and graphs were processed by GraphPad 6 software (GraphPad Software, La Jolla, CA, USA).

## Figures and Tables

**Table 1:** LNA probe-based Real-Time PCR systems.

Gene	5′-Reverse Primer-3′	5′-Forward Primer-3′	#LNA Probe
BACT ^1^	TCCCTGGAGAAGAGCTACGA	AAGAGCGCCTCTGGACAC	9
CYPA ^2^	CCTGAAGCATACGGGTCCT	AAAGACCACATGTTTGCCATC	48
CLD-1 ^3^	CACCACTTTGCAAGCAACC	TGGCCACAAAGATGGCTATT	3
CLD-2 ^4^	CTCGCGCCAAAGACAGAG	ATGAAGATTCCACGCAACG	77
OCLN ^5^	AAAGAGCTCTCTCGACTGGATAAA	AGCAGCAGCCATGTACTCTTC	42
IL-8	TTCTTCTTTATCCCCAAACTGG	CCACATGTCCTCAAGGTAGGA	41
TNF-α	TCAGGGATTCAGGGATGTGT	GAAGCCCCAGTTCCAATTC	77
IL-10	TGCCTCCCACTTTCTCTTGT	TTCCTATGAGTGTAAGCGACTTTG	23

^1^ β-actin, ^2^ cyclophilin A, ^3^ claudin-1, ^4^ claudin-2, ^5^ occludin.

## Appendix A

All the mutations were generated by the one-step PCR inactivation method [56] using the primers and protocol as described previously [17]. After the selection of antibiotic-resistant clones, in which the gene of interest was replaced either with a chloramphenicol or kanamycin gene cassette, the mutation was confirmed by PCR using primers from the sequence flanking the deleted gene sequence in combination with c1 and c2, or k1 and k2 primers [56]. In all the mutants, the chloramphenicol or kanamycin gene cassettes used for the gene replacement were removed from the mutant’s genome.

The Δ*rfa* mutants have been repeatedly characterized and tested for sensitivity to serum, resistance to P22 phage infection, and by PCR using primers both inside and also flanking the deleted sequences. All gradually accumulated data confirmed expected characteristics and therefore the identity of the Δ*rfa* mutants.
